# Determining the impact of professional body recommendations on the screening of acquired carbapenemase-producing Enterobacterales in England

**DOI:** 10.1016/j.infpip.2023.100281

**Published:** 2023-04-01

**Authors:** Kirsty F. Bennet, Rebecca L. Guy, Sarah M. Gerver, Katie L. Hopkins, Richard Puleston, Colin S. Brown, Katherine L. Henderson

**Affiliations:** a Healthcare-Associated Infections, Fungal, Antimicrobial Resistance, Antimicrobial Usage and Sepsis Division, United Kingdom Health Security Agency (UKHSA), London, UK; b Field Services Midlands Regions Directorate, United Kingdom Health Security Agency (UKHSA), Nottingham, UK

**Keywords:** Carbapenemase-producing, Enterobacterales, CPE, Screening, COVID-19 pandemic, Policy

## Abstract

**Introduction:**

Acquired carbapenemase-producing Gram-negative bacteria are an increasing public health concern globally and have been mandatory to report in England since October 2020. However, in light of the COVID-19 (SARS-CoV-2) pandemic, the Royal College of Pathologists (RCPath) released new guidance “for reducing the need for screening of CRE (carbapenem-resistant Enterobacterales) […] in low-risk areas”, without defining “low risk”.

**Methods:**

To assess the impact of the RCPath recommendations on screening of carbapenemase-producing Enterobacterales (CPE), an online Select Survey was sent to all NHS acute hospitals in England. The initial survey distribution was between March and April 2021 and the survey was relaunched between November 2021 and March 2022.

**Results:**

In total, 54 hospitals completed the survey, representing 39.1% of 138 eligible Trusts. All hospitals had a CPE screening policy in place, and the majority of these reflect UKHSA's Framework of actions to contain CPE. Of the 23 hospitals who reported a reduction in CPE screening, only three (13.0%) indicated that this was due to the RCPath recommendations, with 21 (91.3%) indicating that there had been a natural reduction in the number of patients admitted to the Trust who would have previously been screened due to the COVID-19 pandemic.

**Conclusion:**

For most surveyed hospitals, CPE screening was not reduced due to the RCPath recommendations. However, the results highlighted that there is a large amount of individual variation in CPE screening practices and diagnostic testing between hospitals.

## Introduction

Acquired carbapenemase-producing Gram-negative bacteria are an increasing public health concern globally. One of the key deliverables in England's five-year National Action Plan introduced in 2019 is to prevent and control the spread of carbapenemase-producing Enterobacterales (CPE). [1].

In England, the prominent carbapenemase families, known as the ‘big five’ (KPC, NDM, OXA-48-like, VIM and IMP) are currently detected in >97% of all CPE referred to the UK Health Security Agency's (UKHSA) Antimicrobial Resistance and Healthcare Associated Infections (AMRHAI) Reference Unit. [2] To improve our understanding of the local, regional, and national epidemiology of CPE, a key focus has been developing local screening capabilities, particularly the ability for local laboratories to detect the ‘big five’ carbapenemase mechanisms. The national guidance for referral of isolates to the AMRHAI Reference Unit has also changed – while labs are now requested to only send locally confirmed CPE to AMRHAI if isolated from an invasive site, isolates from other clinically relevant sites and from colonised patients are still accepted if laboratories cannot perform testing locally or require confirmation of their results. Laboratories may also have been encouraged to increase local testing when the AMRHAI Reference Unit introduced a charge for testing in April 2018. [3].

To further enhance surveillance, on the 1^st^ October 2020, it became mandatory for all diagnostic laboratories in England to notify UKHSA (formerly Public Health England) of any acquired carbapenemase-producing Gram-negative bacteria identified in human samples, including antimicrobial susceptibility and carbapenemase family results. [4] However, prior to this in March 2020, the Royal College of Pathologists (RCPath) and Professional Bodies (Institute of Biomedical Science [IBMS], the Association of Clinical Biochemistry and Laboratory Medicine [ACB] and the Association of Clinical Pathologists [ACP]) released new screening recommendations in light of the COVID-19 (SARS CoV-2) pandemic. [5] These recommendations were regarding the prioritisation/deferral of pathology laboratory work on the screening of various other organisms. Specifically, for CPE, there was a recommendation “for reducing the need for screening of CRE (carbapenem-resistant Enterobacterales) […] in low-risk areas”, without defining “low risk”. The RCPath guidance was withdrawn in March 2022; however, some residual copies of the guidance document were still accessible online as of 27^th^ November 2022.

The purpose of this study was to determine what impact the RCPath recommendations may have had on the screening of CPE in England from October 2020 onwards.

## Methods

An online impact Select Survey (please refer to Annex 1) was designed by a multidisciplinary UKHSA team to combine elements on screening policy, observed screening numbers and laboratory testing ability. The website link to access the survey online was launched on 31^st^ March 2021 for four weeks and distributed via email to Consultants in Public Health Infection at all NHS acute hospitals in England. Responses were assigned to one of the nine regions using data from the Office for National Statistics (ONS). Due to initial low response rates in certain regions, the survey was relaunched between November 2021 and March 2022 and Infection Prevention and Control colleagues in regions with a low response rate were followed up by UKHSA's Field Service. The survey results were then downloaded into MS Excel. All electronic responses received were independently validated by two of the authors (KFB and KLHe); this included checking data for completeness and duplication, with duplicate responses removed from analysis. Respondents were able to select more than one answer for some questions and could opt not to respond to all questions. Descriptive analysis was performed in MS Excel, and results reflect the percentage of total responses received for each question of the survey.

## Results

**Figure 1:**
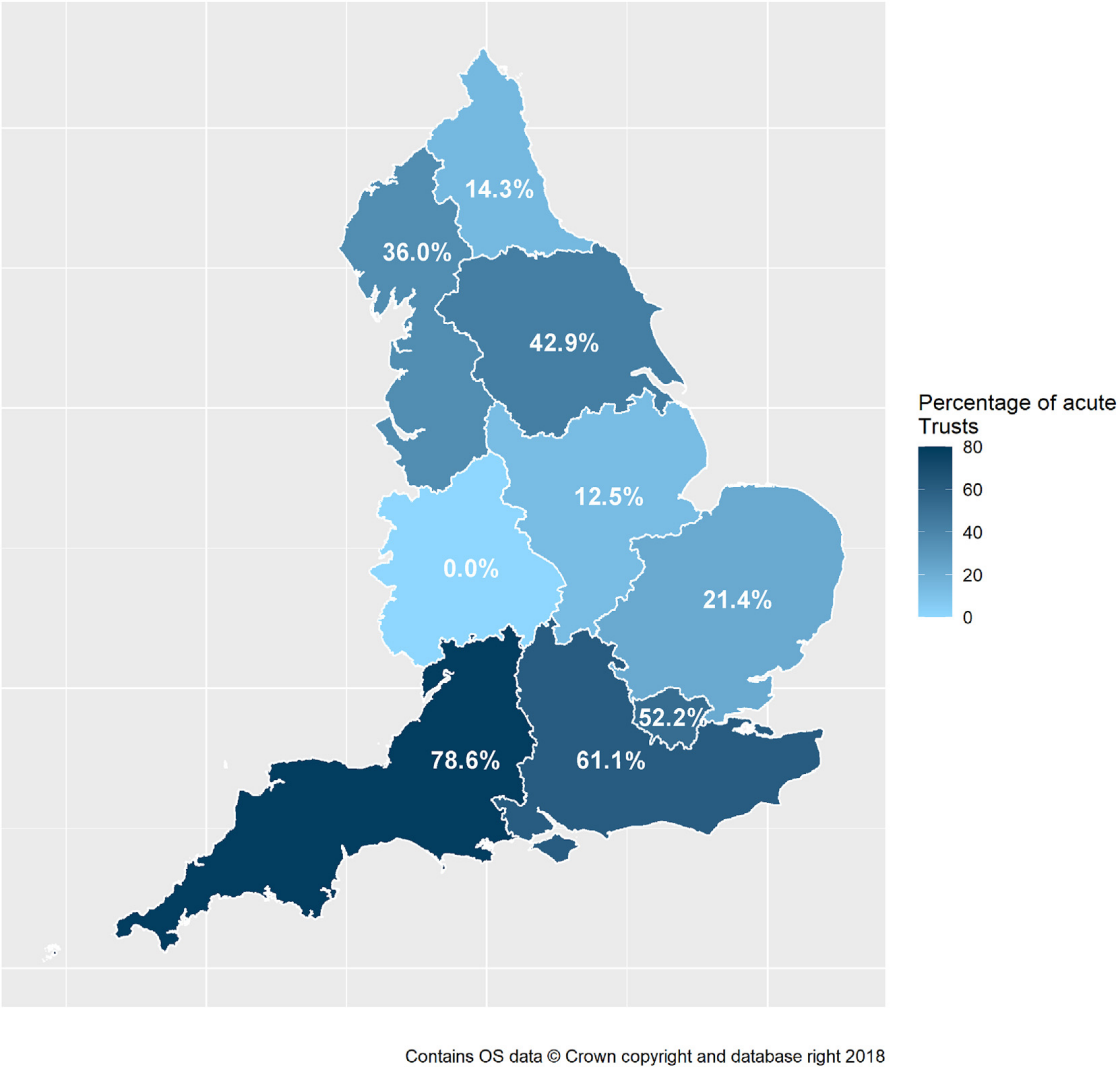
Percentage of total acute Trusts in each of the nine English ONS Regions that completed the survey (n=54).

In total, respondents from 54 different hospitals, each from a different acute NHS Trust, completed the survey (39.1% of 138 eligible Trusts as of September 2021). The initial distribution of the survey in early 2021 returned 46 responses and following the relaunch there were an additional eight responses. Eight of the nine English ONS regions had representation from one or more Trust (Figure 1). The most represented regions were the South West (11 responders; 78.6% of all eligible acute Trusts), South East (11; 61.1%), London (12; 52.2%), and Yorkshire & the Humber (6; 42.9%). Only one response each was received from the North East and East Midlands regions (representing 14.3% and 12.5% of all eligible NHS acute Trusts, respectively). No responses were received from the West Midlands.

**Figure 2:**
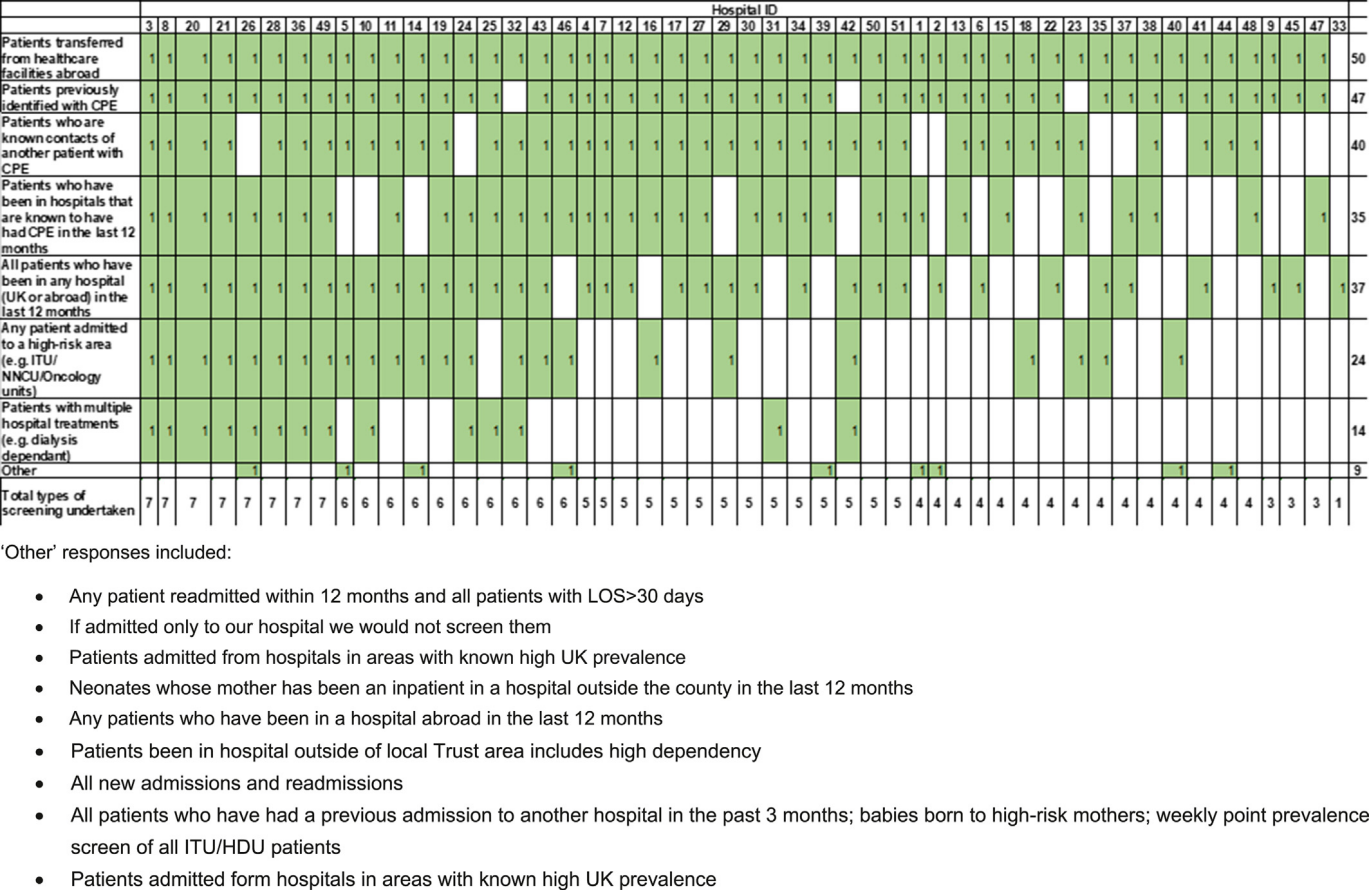
Matrix showing the multi-select responses for which patients each hospital screens for CPE (n=51).

The remainder of the results will be described at a hospital level. Table I highlights the CPE screening policy results; all 54 hospitals indicated that they had a CPE screening policy in place, although this was the only question that all 54 respondents answered. Of the 54 hospitals, 51 provided details of their screening policy (94.4%). Almost half of these (49.0%; n=25/51) indicated that their screening policy had been updated since 2020; however, two respondents indicated that there had been no policy update since 2015 (3.9%). Nearly all responding hospitals indicated that they screen patients transferred from healthcare facilities abroad (98.0%; n=50/51) and patients previously identified with CPE (92.2%; n=47/51). Furthermore, 78.4% (n=40/51) of hospitals reported screening patients that are known contacts of another patient with CPE and 68.6% (n=35/51) of hospitals reported screening all patients who have been in any hospital (UK or abroad) in the last 12 months. The full matrix of answers is shown in Figure 2.

**Table I:** Survey results for Questions 2–9 about to CPE screening policy

Question	Options	Responses	%
Q2. Do you have a screening policy in place for CPE?	Yes	54	100.0
	No	0	0.0
	**Total**	**54**	**100.0**
Q3. When was your screening policy last updated?	2015	2	3.7
	2016	2	3.7
	2017	3	5.6
	2018	9	16.7
	2019	10	18.5
	2020	9	16.7
	2021	16	29.6
	No response	3	5.6
	**Total**	**54**	**100.0**
Q4. Which of the following do you screen for CPE?^a^	Patients transferred from healthcare facilities abroad	50	98.0
	Patients previously identified with CPE	47	92.2
	Patients who are known contacts of another patient with CPE	40	78.4
	Patients who have been in hospitals that are known to have had CPE in the last 12 months (including ITUs, transplant units, SCBUs, oncology units)	35	68.6
	All patients who have been in any hospital (UK or abroad) in the last 12 months	37	72.5
	Any patient admitted to a high-risk area (e.g. ITU/NNCU/Oncology units)	24	47.1
	Patients with multiple hospital treatments (e.g. dialysis dependant)	14	27.5
	Other a	9	17.6
	**Total**	**51**	**100.0**
Q5. What is your screening regimen?^a^	1 admission screen	24	47.1
	3 admission screens 48-hours apart	23	45.1
	Weekly screening in high-risk areas (e.g. ITU, NNIC, Oncology units)	12	23.5
	Monthly screening in high-risk areas	3	5.9
	Other	13	25.5
	**Total**	**51**	**100.0**
Q6. Does the Trust's CPE screening policy reflect PHE's Action to contain CPE? (Published in October 2020)	Yes	28	56.0
	No^b^	22	44.0
	Currently under review	7	14.0
	Partially	5	10.0
	**Total**	**50**	**100.0**
Q7. Does this policy differ across the Trust and/or in different areas of the hospital (aside the previous sections outlined in the screening regime?	No	50	100.0
	Yes	0	0.0
	**Total**	**50**	**100.0**
Q8. Has there been a reduction in CPE screening?	Yes	23	45.1
	No	28	54.9
	**Total**	**51**	**100.0**
Is this reduction due to?	a) A change in screening policy for CPE following the RCPath recommendations	3^c^	13.0
	b) A natural reduction in the number of patients admitted to hospital who would have previously been screened due to the COVID-19 pandemic	21^c^	91.3
	c) Capacity or resourcing constraints	2^c^	8.7
	**Total**	**23**	**100.0**
Q9. If CPE screening has been reduced due to 8b) or 8c), when do you think your Trust will be able to restore its CPE screening policy?	Do not think will restore	1	7.7
	In the next 3 months	12	66.7
	In the next year	5	29.4
	**Total**	**18**	**100.0**

a Respondents were able to select multiple options for this question, please see Figure 2, Figure 3.

b Of the respondents who answered ‘No’, 7 indicated that their screening policy was currently under review or being updated, and 5 indicated that their screening policy partially reflected these guidelines.

c 1 responder said yes to a), b) and c); 1 responder said yes to answers a) and b).

Fifty-one responders provided information on their CPE screening regimen; the variety of responses are shown in Figure 3. The most common practices for CPE screening were one admission screen (47.1%; n=24/51), three admission screens 48-hours apart (45.1%; n=23/51) and weekly screening in high-risk areas, for example Intensive Care Units (ICU) and Oncology units (23.5%; n=12/51) (Table I). All responders indicated that there were no additional differences in screening policy across their Trust.

**Figure 3:**
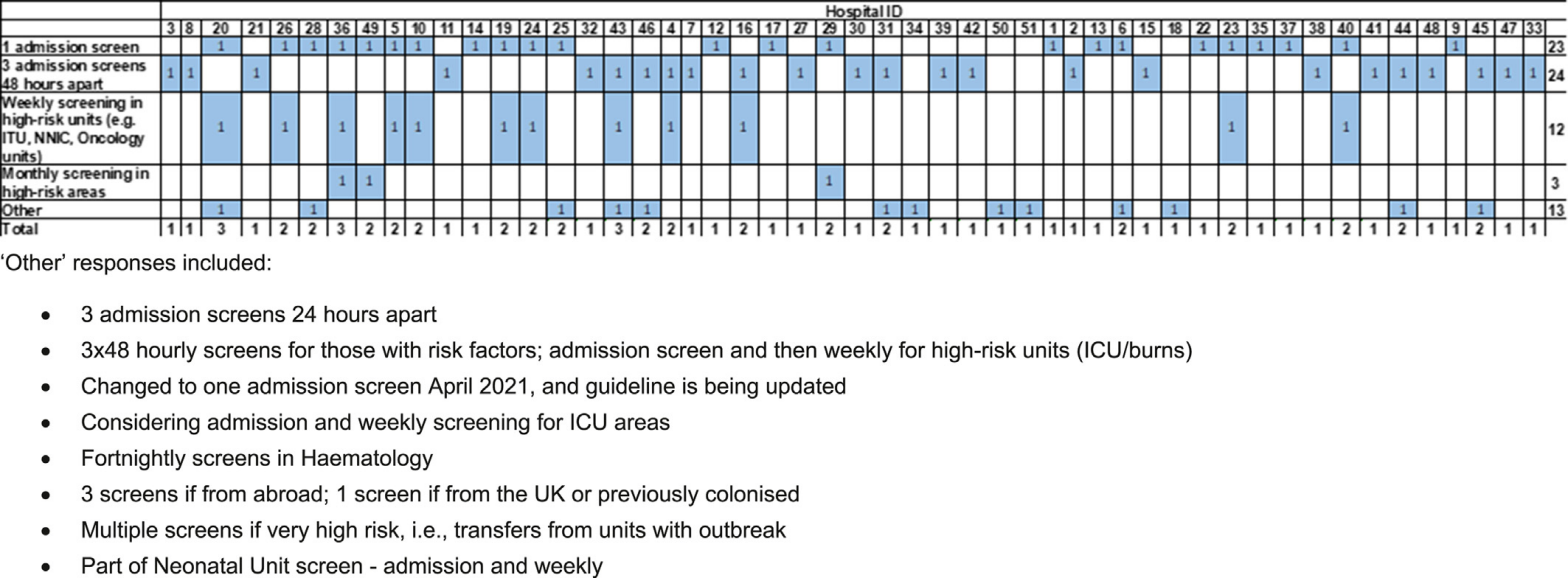
Matrix showing the multi-select responses for each hospital's screening regimen (n=51).

When asked whether there had been a reduction in screening during 2020, 28 hospitals reported no reduction (51.9%), 23 responders (42.6%) indicated that there had been a reduction and three (5.6%) did not respond. Of those who noted a reduction in CPE screening, three hospitals (13.0%; n=3/23) indicated that this was due to the RCPath recommendations. For two hospitals, the RCPath recommendations were the only reason for a reduction in screening, and for one hospital this reduction was also due to a natural reduction in the number of patients admitted to the hospital as well as capacity and resourcing constraints. Most hospitals (91.3%; n=21/23) indicated that there had been a natural reduction in the number of patients admitted to the hospital who would have previously been screened, due to the COVID-19 pandemic, of which two (9.5%; n=2/21) reported that capacity and resourcing constraints also reduced CPE screening.

**Figure 4:**
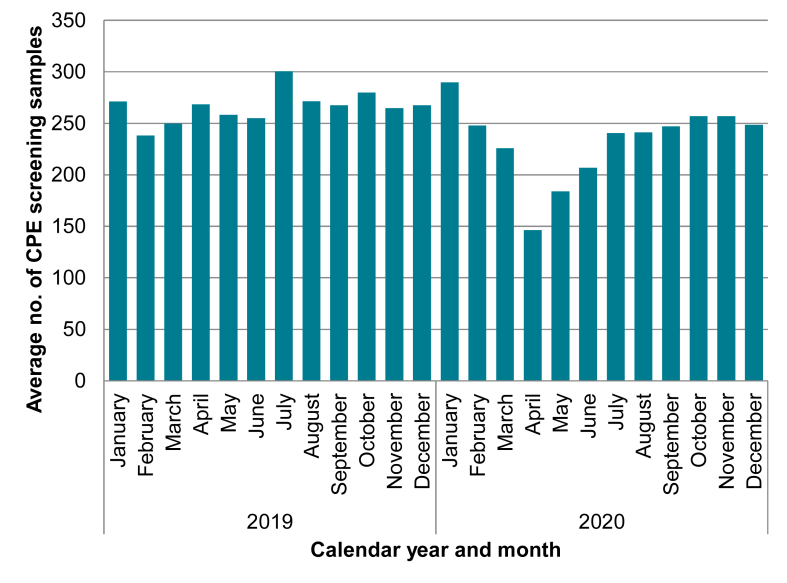
The average number of monthly CPE screening swabs performed by each hospital, 2019–2020 (n=34).

Hospitals were also asked how many screening swabs were taken each month in 2019 and 2020. Thirty-four hospitals provided a complete or almost complete response (missing ≤3 totals across both 2019 and 2020), and seven hospitals provided complete totals for 2020 only. Looking only at complete responses, there was a 35.1% decrease in the average screening swabs taken across all hospitals between March and April 2020 (from 225.7 to 146.4 average swabs; Figure 4), aligning with the peak of the COVID-19 pandemic ‘first wave’ in England. From May 2020 onwards, the average number of CPE screening swabs performed across all hospitals rose, but it had not recovered back to pre-pandemic levels as of December 2020. The monthly average number of swabs taken per hospital across 2019 was 265.2, whereas in 2020 this was 232.3 swabs, a 12.4% reduction.

Cross-checking the data between a hospital that indicated they had observed a decrease in screening activities and the actual number of screens provided, 84.2% of hospitals who answered that they had observed a reduction (n=16/19) also had a true reduction in their average screens from 2019 to 2020. However, in two hospitals the average screening counts remained stable, and one saw a three-fold increase in the number of screening swabs performed, contrary to the respondent's opinion. The screening swab numbers provided were at the acute Trust level in three quarters of responses (75.0%; n=27/36) and for the hospital level only for 25.0% of responses (n=9/36).

Table II shows that the majority (88.0%) of responding hospitals can detect the ‘big four’ carbapenemase families (KPC, OXA-48-like, NDM and VIM) and most could also detect IMP (84.0%; n=42/50). However, there were three hospitals who did not have the ability to detect any of the ‘big five’ carbapenemase families listed and used either another local laboratory or sent it to a regional UKHSA laboratory or the AMRHAI Reference Unit, and one hospital where this information was unknown.

**Table II:** The carbapenemase family(ies) that the hospitals are able to detect (n=50)

Question	Option	Responses	%
Which carbapenemase family(ies) is your laboratory able to detect?	KPC	46	92.0
	NDM	45	90.0
	OXA-48-like	45	90.0
	VIM	44	88.0
	IMP	42	84.0
	None^a^	3	6.0
	Unknown	1	2.0
	**Total**	**50**	**100.0**

a Of these three hospitals, one tested only for meropenem resistance and then sent the isolate to the AMRHAI Reference Unit, and another used either a different local laboratory or sent it to a regional UKHSA laboratory.

For each of the ‘big five’ carbapenemase families, the most common method of detection was the Xpert® Carba-R (Cepheid) PCR assay (which was used by over 56.3% of hospitals for each carbapenemase family), followed by the NG-Test CARBA 5 (NG- Biotech) immunochromatographic assay (used by over 15.2% of hospitals for each carbapenemase family; Table III). Although the Xpert® Carba-R assay can be used to detect all of the ‘big five’ carbapenemases, 56.3% (n=27/48) of responding hospitals use it to detect KPC whereas 64.1% (n=25/39) of responding hospitals use it to detect IMP. These testing capabilities were in-house in most cases, meaning that samples did not need to be referred to another local/regional/national laboratory (84.6%; n=44/52).

**Table III:** The assay detection method used for the ‘big 5’ carbapenemase families (n=48)

For the carbapenamases above, what method(s) do you use to detect these? (n=46)	KPC	%	NDM	%	OXA-48-like	%	VIM	%	IMP	%
Cepheid: XPERT® CARBA-R	27	56.3	26	56.5	26	56.5	26	61.9	25	64.1
Coris: RESIST– 4 O.K.N.V.	2	4.2	2	4.3	2	4.3	1	2.4	0	0.0
Coris: RESIST– 5 O.K.N.V.I.	4	8.3	4	8.7	4	8.7	4	9.5	3	7.7
Coris: RESIST– 6 O.O.K.N.V.I.	1	2.1	1	2.2	1	2.2	1	2.4	1	2.6
NG-Biotech: NG-test Carba 5	8	16.7	7	15.2	7	15.2	7	16.7	7	17.9
Other	6	12.5	6	13.0	6	13.0	3	7.7	3	7.7
**Total**	**48**	**100.0**	**46**	**100.0**	**46**	**100.0**	**42**	**100.0**	**39**	**100.0**

## Discussion

This survey describes the status of CPE screening in England in 2019 and 2020, the impact of the COVID-19 pandemic on local activity, and compliance with the RCPath recommendations.

Our survey demonstrates that having a CPE screening policy in place is a common occurrence, and, from the survey respondents, the majority of CPE policies reflect the UKHSA's Framework of actions to contain CPE (one admission screen) or were in the process of being updated to reflect it. [6].

Almost half (45.1%) of hospitals reported that there had been a reduction in CPE screening but only 13.0% indicated that this was due to the RCPath recommendations. This suggests that the RCPath recommendations did not substantially affect the overall screening of CPE in these hospitals and that the guidelines' impact was largely overshadowed by a reduced number of patients being admitted to NHS hospitals for non-COVID-19 care during the COVID-19 pandemic in 2020. A reduction in patient numbers, changes in patient mix and subsequent incidence reduction for endogenous pathogens over the early COVID-19 pandemic has been reported elsewhere. [7,8].

Although the survey did not capture this information, it would be helpful to discern the level of implementation of the RCPath guidance considered by hospitals. For example, would the reduction in CPE screening have been seen even if the reduction in patients due to COVID-19 did not occur? In addition, it would be interesting to assess if and/or how this view changed over the course of the COVID-19 pandemic.

Of the hospitals that had seen a reduction in CPE screening, almost all (>90%) predicted that this would restore to normal levels within the year following the survey. This may be because the number of patients in hospitals was still reduced during 2020 as the COVID-19 pandemic continued. Over the course of 2021, as the UK began a phased exit from lockdown and restrictions eased, NHS hospital admissions rose and exceeded those seen in 2020. [9] An important future extension to this work would be to assess how increasing patient numbers impacted CPE screening across 2021 and 2022, particularly after the withdrawal of the RCPath recommendations.

Another important finding is that most hospitals (88.2%; n=44/51) are able to detect the ‘big four’ carbapenemase families using either PCR or immunochromatographic methods, which is in line with UKHSA's recommendation that all diagnostic laboratories should be able to do so. [6,10] However, all of the carbapenemase assays used by hospitals are able to detect the ‘big four’ carbapenemases, so it is unclear why two laboratories responded to say that they could not, as shown by the lower percentages of laboratories able to detect OXA-48-like, NDM and VIM reported in Table III.

The low response rates, especially from the West Midlands that did not have any responders, may introduce reporting bias. Similarly, only one response each was received from both the North East and East Midlands regions. Although the large amount of variation seen in survey responses is a limitation of this study, this highlights the need for further work to look at regional reporting of CPE.

The findings of this survey demonstrate that CPE data were not dramatically impacted by the RCPath recommendations, which is important in the interpretation of UKHSA data outputs on CPE since October 2020. [2,11] Another key finding is that there is a great deal of individual variation in CPE screening practices and diagnostic testing between hospitals. To further contextualise CPE positive cases from background screening data, UKHSA subsequently performed a CPE point prevalence survey in ICUs in April 2022, and analysis of the results is currently underway. Highlighting the differences in screening practice may help to inform other acute Trusts on what they could do to help reduce the spread of CPE which would help to work towards the 5-year National Action Plan for AMR. [1].

## Credit author statement

**Kirsty F Bennet**: Methodology, Software, Validation, Formal analysis, Investigation, Resources, Data curation, Writing – original draft, Visualization. **Rebecca L Guy**: Methodology, Validation, Writing – review and editing, Project administration. **Sarah M Gerver**: Conceptualization, Methodology, Writing – review and editing, Supervision. **Katie L Hopkins**: Writing – review and editing. **Richard Puleston**: Investigation, Writing – review and editing. **Colin S Brown**: Conceptualisation, Methodology, Writing – review and editing, Supervision. **Katherine L Henderson**: Conceptualization, Methodology, Writing – review and editing, Project Administration.

## Acknowledgements

We would like to thank hospital and laboratory staff who took the time to respond to the questionnaire.

## Conflict of interest statement

None declared.

## Funding source

This work was carried out as part of the routine work of the UK Health Security Agency (UKHSA); no additional funding or grants were received.
