# Supplementary material for: Determining the impact of professional body recommendations on the screening of acquired carbapenemase-producing Enterobacterales in England
Source: Infect Prev Pract. 2023 Apr 1;5(2):100281. doi: 10.1016/j.infpip.2023.100281 (PMC10160507; doi:10.1016/j.infpip.2023.100281)
Supplement: Multimedia component 1 [file mmc1.docx]

**Annex 1: Survey to find out the impact of RCPath’s Prioritisation/deferral of Pathology Laboratory Work on CPE screening (in light of SARS CoV-2 epidemic) from March 2020 onwards**

1. Please enter your hospital and trust name
   **[Hospital name]
   [Trust name]**
2. Do you have a screening policy in place for CPE?

- Yes
- No

***If no go to Question 8; if YES go to Question 3:***

1. When was your policy last updated? **[MM/YYYY]**
2. Which of the following do you screen for CPE?

- All patients who have been in any hospital (UK or abroad) in the last 12 months
- Patients who have been in hospitals that are known to have had CPE in the last 12 months (including ITUs, transplant units, SCBUs, oncology units)
- Patients transferred from healthcare facilities abroad
- Patients previously identified with CPE
- Patients who are known contacts of another patient with CPE
- Patients with multiple hospital treatments (e.g. dialysis dependant)
- Any patient admitted to a high-risk area (e.g. ITU/ NNCU/Oncology units)
- Other, please specify **[free text]**

1. What is your screening regimen?

- 1 admission screen
- 3 admission screens 48-hours apart
- Weekly screening in high-risk areas (e.g. ITU, NNIC, Oncology units)
- Monthly screening in high-risk areas
- Other, please specify **[free text]**

1. Does the trust’s CPE screening policy reflect PHE’s Action to contain CPE? (published in October 2020) <https://www.gov.uk/government/publications/actions-to-contain-carbapenemase-producing-enterobacterales-cpe> (PHE’s Action to contain CPE begins on page 13)

- Yes
- No
- Other, please specify **[free text]**

1. Does this policy differ across the Trust and/or in different areas of the hospital(s)?

- No
- Yes, please specify **[free text]**

1. Has there been a reduction in CPE screening due to either:

|  | **Yes, a reduction** | **No** |
| --- | --- | --- |
| a) A change in screening policy for CPE following the RCPath recommendations published in March 2020 that included [reducing the need for screening of CRE (Carbapenem-Resistant Enterobacteria) and VRE (Vancomycin-Resistant Enterococcus) in low risk areas](https://www.rcpath.org/uploads/assets/f5123842-950f-49c5-bf69ed866a7ca3da/Prioritisation-deferral-of-pathology-laboratory-work.pdf)? | □ | □ |
| b) A natural reduction in the number of patients admitted to hospital who would have previously been screened as a result of the COVID-19 pandemic? | □ | □ |
| c) Capacity or resourcing constraints? | □ | □ |

1. If CPE screening has been reduced due to 8b) or 8c), when do you think your Trust will be able to restore its CPE screening policy?

- Do not think will restore
- In the next 3 months
- In the next year

1. How many CPE screening swabs were taken each month in 2019 and 2020?

|  | **Jan** | **Feb** | **Mar** | **Apr** | **May** | **Jun** | **Jul** | **Aug** | **Sep** | **Oct** | **Nov** | **Dec** |
| --- | --- | --- | --- | --- | --- | --- | --- | --- | --- | --- | --- | --- |
| **2019** |  |  |  |  |  |  |  |  |  |  |  |  |
| **2020** |  |  |  |  |  |  |  |  |  |  |  |  |

1. Are these screening swab numbers for your trust or for your hospital only?

- Trust level
- Hospital only

1. Please tick the resistance mechanism(s) your laboratory is able to detect (please select all that apply):

- KPC
- OXA-48-like
- NDM
- VIM
- IMP
- Other, please specify **[free text]**

1. For the carbapenamases ticked above, what method(s) do you use to detect these – please provide precise assay/kit details for each:

| \|  \| **KPC** \| **OXA-48-like** \| **NDM** \| **VIM** \| **IMP** \| **Other** \| \| --- \| --- \| --- \| --- \| --- \| --- \| --- \| \| Cepheid: XPERT® CARBA-R \| □ \| □ \| □ \| □ \| □ \| □ \| \| Coris: RESIST– 4 O.K.N.V. \| □ \| □ \| □ \| □ \| □ \| □ \| \| Coris: RESIST– 5 O.K.N.V.I. \| □ \| □ \| □ \| □ \| □ \| □ \| \| Coris: RESIST– 6 O.O.K.N.V.I. \| □ \| □ \| □ \| □ \| □ \| □ \| \| NG-Biotech: NG-test Carba 5 \| □ \| □ \| □ \| □ \| □ \| □ \| \| Other \| □ \| □ \| □ \| □ \| □ \| □ \| |
| --- | --- | --- | --- | --- | --- | --- | --- | --- | --- | --- | --- | --- | --- | --- | --- | --- | --- | --- | --- | --- | --- | --- | --- | --- | --- | --- | --- | --- | --- | --- | --- | --- | --- | --- | --- | --- | --- | --- | --- | --- | --- | --- | --- | --- | --- | --- | --- | --- | --- |

1. Are these testing capabilities in-house or do you refer these to another regional laboratory?

- In-house
- Referred to regional lab

1. Based on the above testing, when do you decide to refer isolates to the Colindale reference laboratory (AMRHAI)?
   **[free text]**
2. Please could you supply a named Consultant microbiologist who is happy to act as a point of contact for PHE HCAI & AMR enquiries?
   **[Name]
   [Email address]
   [Contact number]**
3. Are you happy for us to share the contact information provided in Question 16 with NHSE’s HCAI lead?

- Yes
- No
